# Norovirus Outbreak Surveillance, China, 2016–2018

**DOI:** 10.3201/eid2603.191183

**Published:** 2020-03

**Authors:** Miao Jin, Shuyu Wu, Xiangyu Kong, Huaping Xie, Jianguang Fu, Yaqing He, Weihong Feng, Na Liu, Jingxin Li, Jeanette J. Rainey, Aron J. Hall, Jan Vinjé, Zhaojun Duan

**Affiliations:** Chinese Center for Disease Control and Prevention, Beijing, China (M. Jin, X. Kong, N. Liu, J. Li, Z. Duan);; US Centers for Disease Control and Prevention, Beijing (S. Wu, J.J. Rainey);; Guangzhou Center for Disease Control and Prevention, Guangzhou, China (H. Xie);; Jiangsu Provincial Center for Disease Control and Prevention, Nanjing, China (J. Fu);; Shenzhen Center for Disease Control and Prevention, Shenzhen, China (Y. He);; Wuxi Center for Disease Control and Prevention, Wuxi, China (W. Feng);; US Centers for Disease Control and Prevention, Atlanta, Georgia, USA (A.J. Hall, J. Vinjé)

**Keywords:** Norovirus, outbreak, surveillance, China, viruses, acute gastroenteritis, enteric infections

## Abstract

CaliciNet China, a network of provincial, county, and city laboratories coordinated by the Chinese Centers for Disease Control and Prevention, was launched in October 2016 to monitor the epidemiology and genotype distribution of norovirus outbreaks in China. During October 2016–September 2018, a total of 556 norovirus outbreaks were reported, and positive fecal samples from 470 (84.5%) outbreaks were genotyped. Most of these outbreaks were associated with person-to-person transmission (95.1%), occurred in childcare centers or schools (78.2%), and were reported during November–March of each year (63.5%). During the 2-year study period, 81.2% of all norovirus outbreaks were typed as GII.2[P16]. In China, most norovirus outbreaks are reported by childcare centers or schools; GII.2[P16] is the predominant genotype. Ongoing surveillance by CaliciNet China will provide information about the evolving norovirus genotype distribution and outbreak characteristics important for the development of effective interventions, including vaccines.

Human noroviruses are the leading cause of outbreaks of acute gastroenteritis, associated with ≈50% of all outbreaks worldwide (*1*). Norovirus outbreaks are frequently reported in semiclosed institutions, such as hospitals, nursing homes, schools, and childcare centers (*2*). The virus is primarily transmitted directly from person to person or indirectly through contaminated surfaces, food, or water (*1*). The relative stability of noroviruses on environmental surfaces makes infection control challenging (*3*). Several candidate norovirus vaccines are in clinical trials (*4*).

Noroviruses are single-stranded RNA viruses that belong to the genus *Norovirus*, family *Caliciviridae*. The genome is organized into 3 open reading frames (ORFs): ORF1 encodes polyprotein, ORF2 encodes the major capsid protein (VP1), and ORF3 encodes the minor (VP2) capsid protein. The viruses are classified into at least 7 genogroups (G), of which viruses from GI, GII, and GIV infect humans (*5*,*6*). On the basis of the diversity of VP1, these genogroups can be further divided into at least 33 genotypes: 9 GI, 22 GII, and 2 GIV (*7*). In addition, on the basis of the diversity of the polymerase region of ORF1, >14 GI polymerase (GI.P) types and 27 GII.P types have been described (*7*). Because of the frequent recombination at the ORF1/ORF2 junction region, a dual-typing system has been proposed for GI and GII noroviruses (*7*). Since 2002, genogroup II, genotype 4 (GII.4), noroviruses have been associated with most norovirus outbreaks globally, and new GII.4 variants have emerged every 2–3 years (*8*). Monitoring the trends in the distribution of the various genotypes and possible association of certain strains with a more severe disease outcome is important for understanding and controlling norovirus epidemics (*9*).

Several norovirus outbreak surveillance networks, including NoroNet (*10*) and CaliciNet (*7*,*11*), have been developed during the past decade. NoroNet captures molecular and epidemiologic data on norovirus outbreaks and sporadic cases submitted by 19 participating countries across Europe and Asia and by Australia. CaliciNet is a norovirus outbreak surveillance network in the United States in which state and local public health laboratories electronically submit laboratory data, including sequences from norovirus outbreaks, to a central database (https://www.cdc.gov/norovirus/reporting/calicinet/data.html). CaliciNet data are integrated with epidemiologic data from the National Outbreak Reporting System, yielding comprehensive surveillance in the United States that enables multifactorial characterization of norovirus outbreaks (*9*).

Since 2004, provincial and local Centers for Disease Control and Prevention (CDCs) in China have been required to report all acute gastroenteritis outbreaks of >20 cases, including those caused by noroviruses, to a passive national outbreak surveillance system called Public Health Emergency Event Surveillance System (PHEESS) (*12*). Although PHEESS provides useful information, detailed epidemiologic and molecular data from norovirus outbreaks are typically not included. In October 2016, the National Institute for Viral Disease Control and Prevention at the China CDC launched CaliciNet China as a surveillance network to monitor the epidemiology and molecular characteristics of norovirus outbreaks. We describe this new network and report data from the first 2 years of surveillance.

## Materials and Methods

### CaliciNet China

For the surveillance of norovirus outbreaks, CaliciNet China relies on contributions from laboratory and epidemiologic staff at county, city, and provincial CDCs. County CDCs are responsible for acute gastroenteritis outbreak investigations and collection of clinical specimens. Laboratories of city and provincial CDCs perform norovirus detection and genotyping on the specimens. The national laboratory at China CDC receives and aggregates findings and provides overall quality control. Laboratory staff at these CDCs received training on using the standardized methods of norovirus detection and typing (*7*). Each laboratory performs proficiency testing once a year for quality assurance.

### Outbreak Reporting

CaliciNet China defines norovirus outbreaks as >5 acute gastroenteritis cases within 3 days after exposure in a common setting where >2 samples (whole fecal, rectal swab, or vomitus) had been laboratory confirmed as norovirus. Acute gastroenteritis was defined as >3 events involving loose feces, vomiting, or both within a 24-hour period. The data were collected in collaboration with the local epidemiologists investigating the outbreaks using national guidelines (*13*). Local CDCs aggregated epidemiologic data for each outbreak, including date of first illness onset, setting, transmission route, number of cases, and type of specimens collected. All data were entered into Microsoft Excel (Microsoft, https://www.microsoft.com). City, county, and provincial CDCs also submitted laboratory results from real-time reverse transcription PCR (rRT-PCR), conventional RT-PCR, and sequences into a BioNumerics version 6.6 database (Applied Maths**,** *https://www.***applied-maths.***com*) using CaliciNet scripts provided by CaliciNet USA (*7*). Each month, data were sent electronically to China CDC.

### rRT-PCR and Genotyping

For fecal samples, laboratories prepared a 10% fecal suspension by mixing 0.1 g feces with 1.0 mL phosphate-buffered saline (pH 7.2). For swab or vomitus samples, nucleic acid was extracted directly. Laboratories tested viral RNA for GI and GII norovirus using the Ag-Path kit (Applied Biosystems, https://www.fishersci.com) (*7*,*14*) in a duplex rRT-PCR with primers (Cog1F, Cog1R, Cog2F, and Cog2R) (*11*) and TaqMan probe (Ring 1E and Ring 2) (*14*,*15*). The cycling conditions were described previously (*7*). Some laboratories also used commercial norovirus rRT-PCR kits (BioPerfectus Technology Co., http://www.s-sbio.net; Da An GENE Co., http://en.daangene.com). Norovirus-positive samples were then amplified by conventional RT-PCR, which amplifies a partial region of ORF1 and a partial region of ORF2 (*7*,*16*). The RT-PCR conditions and the primers used (MON432, G1SKR, MON431, and G2SKR) have been described previously (*7*,*16*).

### Data Management and Analysis

We merged epidemiologic and genotype outbreak data at China CDC and then imported them into SPSS Statistics 25 software (IBM, https://www.ibm.com) for data cleaning and analysis. We generated descriptive statistics on reported outbreaks by setting, transmission route, outbreak size, and province and examined differences between genotypes and mode of transmission, outbreak size, setting, and season using χ^2^ tests. We used an α value of p<0.05 to assess statistical significance. Genotypes were assigned by phylogenetic analysis using the UPGMA method with reference sequences used by CaliciNet for capsid typing.

### Ethics Review

The China CDC Ethical Review Committee approved CaliciNet China as routine surveillance for norovirus. Because CaliciNet China collects only aggregate data on norovirus outbreaks, we did not analyze any personal identifying information as part of this project. Linkage of specimens to patient name was maintained by provincial-level CDCs and not submitted into the CaliciNet China database.

## Results

### Epidemiologic Characteristics of Norovirus Outbreaks

**Figure 1:**
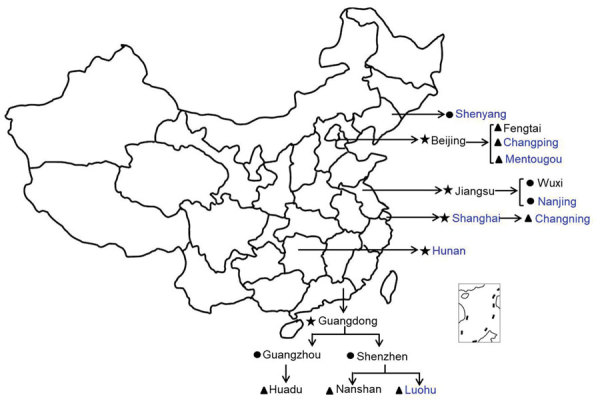
Geographic location of participating local Centers for Disease Control and Prevention in CaliciNet China, October 2016–September 2018. Star indicates provincial/municipality laboratories; circle, city laboratories; triangle, district/county laboratories. Laboratories that participated in CaliciNet China: black, April 2016; blue, April 2017.

**Figure 2:**
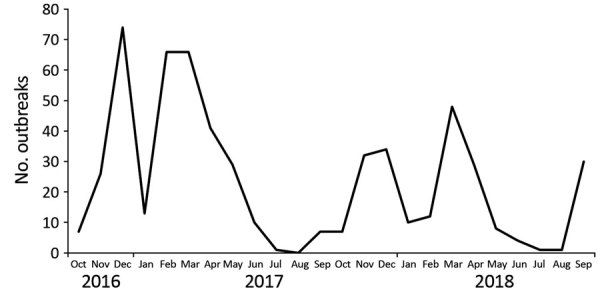
Monthly distribution of norovirus outbreaks reported to CaliciNet China, October 2016–September 2018.

In 2016, CaliciNet China started with 9 laboratories in 3 provinces (Beijing, Guangdong, and Jiangsu) and by 2018 increased to 17 laboratories in 6 provinces (adding Shanghai, Hunan, and Liaoning) distributed across eastern, central, and western China (Figure 1). During October 2016–September 2018, a total of 556 norovirus outbreaks were reported to CaliciNet China. Of these, 320 (57.6%) were reported from Guangdong Province, 101 (18.2%) from Jiangsu Province, 79 (14.2%) from Beijing, 38 (6.8%) from Hunan Province, 12 (2.2%) from Liaoning Province, and 6 (1.1%) from Shanghai. Most outbreaks occurred during the winter season (November–March) (Figure 2). Information about the outbreak size was reported for 427 (76.7%) outbreaks, among which the median size was 15 persons (interquartile range 12–81.5) per outbreak. Five outbreaks each had >200 cases. Three of those occurred in universities and 2 in vocational schools; 4 were caused by person-to person transmission and 1 by foodborne transmission (Table 1). Of the 556 outbreaks, 280 (50.4%) occurred in childcare centers; 155 (27.9%) occurred in primary schools, 61 (11.0%) in middle schools, and 24 (4.3%) in universities (Appendix Table). Of 452 (81.3%) outbreaks in which transmission mode was identified, person-to-person transmission predominated (430 [95.1%]), followed by foodborne transmission (13 [2.9%]) and waterborne transmission (6 [1.3%]) (Table 2).

**Table 1:** Genotype of cases in 556 norovirus outbreaks reported to CaliciNet China, October 2016–September 2018

Genotype	No. outbreaks with known size	No. cases or range	Median no. cases
GII.1[P33]	1	43	43
GII.2[P2]	1	15	15
GII.2[P16]	262	3–236	18
GII.3[P12]	21	3–72	7
GII.4 Sydney[P31]	7	3–50	43
GII.6[P7]	12	4–72	12
GII.8[P8]	2	11–13	12
GII.13[P21]	1	14	14
GII.14[P7]	0	0	0
GIX.1[P15]	1	17	17
GII.17[P17]	14	3–360	33
GII.17[P31]	2	6	6
GII untypeable	11	7–115	15
GI.1[P1]	0	0	0
GI.2[P2]	11	5–122	31
GI.3[P13]	5	5–72	16
GI.5[P12]	1	5	5
GI.6[P11]	5	6–117	30
Multiple genotypes	11	5–348	60
Not determined*	59	3–106	10
Total	427	3–360	15

*The genotyping reverse transcription PCR for these outbreaks was not conducted by network laboratories.

**Table 2:** Number of norovirus outbreaks reported to CaliciNet China for each transmission route and outbreak setting, October 2016–September 2018

Setting	Person-to-person	Foodborne	Waterborne	Unknown	Total
Childcare center	227	2	0	51	280
Primary school	121	3	0	31	155
Middle school	40	3	5	13	61
University	15	3	1	5	24
Company	7	1	0	1	9
Restaurant	2	0	0	2	4
Hospital	3	0	0	0	3
Hotel	1	0	0	1	2
Party	0	0	0	1	1
Multiple school types*	14	1	0	1	16
Unknown	0	0	0	1	1
Total	430	13	6	107	556

*Fifteen outbreaks occurred in schools that combine primary school and middle school, and 1 outbreak occurred in a school that combines a childcare center, a primary school, and a middle school.

### Genotypes

**Figure 3:**
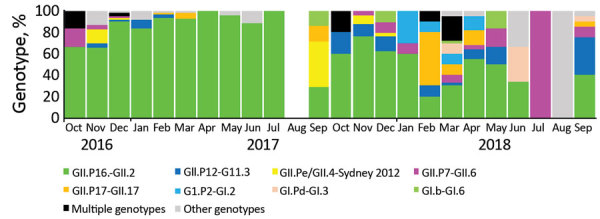
Genotype distribution of 470 norovirus outbreaks reported to CaliciNet China, October 2016–September 2018. No outbreaks were reported in August 2017.

We obtained genotype information for 470 (84.5%) of the 556 outbreaks. Of the 86 (15.5%) outbreaks with no genotyping results, 72 were not further genotyped by network laboratories and 14 were positive by rRT-PCR but negative by conventional RT-PCR and thus could not be genotyped. Of the typed outbreaks, 430 (91.5%) were GII, 26 (5.5%) were GI, and 14 (2.5%) comprised both GI- and GII-positive samples. Overall, 5 GI genotypes and 12 GII genotypes were detected (Appendix Table). Of 470 genotyped outbreaks, GI genotypes included GI.2[P2] (11 [2.3%]), GI.6[P11] (6 [1.3%]), and GI.3[P13] (5 [1.1%]). Among GII outbreaks, 349 (74.3%) were typed as GII.2[P16], which was detected throughout the study period and peaked during winter 2016–17 (Figure 3). Other GII genotypes were GII.3[P12] (25 [5.3%]), GII.17[P17] (18 [3.8%]), GII.6[P7] (16 [3.4%]), and GII.4 Sydney[P31] (11 [2.3%]) (Appendix Table). Genotypes detected in <1% of the outbreaks were GI.1[P1], GI.3[P13], GI.3[P13], GI.5[P12], and GI.6[P11] among GI viruses and GII.1[P33], GII.2[P2], GII.8[P8], GII.13[P21], GII.14[P7], GIX.1[P15], and GII.17[P31] among GII viruses. In the GII.2[P16] epidemic during winter 2016–17, GI viruses were rarely detected (1 [0.3%] of 298), whereas during the following seasons (2017–2018), GI viruses were detected in 24 (13.9%) of 172 outbreaks and peaked in March 2018 (10 [40.0%] of 25). Outbreaks caused by multiple genotypes were mainly detected in March 2018 (9 [64.3%] of 14); GII.17[P17] viruses were mainly detected during January–April 2018 (9 [64.3%] of 14); and GII.6[P7] viruses were detected primarily in the 2017–18 winter season (13 [81.3%] of 16).

### Genotypes Associated with Epidemiologic Characteristics

Among the GII.2[P16] outbreaks, foodborne transmission was reported for 10 (3.6%) of 276 and person-to-person transmission for 266 (96.4%) of 276; no waterborne outbreaks were reported (χ^2^ 10.5; p = 0.003) (Table 3). Outbreaks with multiple genotypes were more often associated with waterborne and foodborne transmission than were outbreaks with a single genotype.

**Table 3:** Transmission routes of genotypes in norovirus outbreaks reported to CaliciNet China, October 2016–September 2018

Genotype	Person-to-person	Foodborne	Waterborne	Unknown	Total
GII.1[P33]	1	0	0	0	1
GII.2[P16]	266	10	0	73	349
GII.2[P2]	1	0	0	1	2
GII.3[P12]	20	0	0	5	25
GII.4 Sydney[P31]	6	0	0	4	10
GII.Pe-GII.4	1	0	0	0	1
GII.6[P7]	11	1	0	4	16
GII.8[P8]	2	0	0	0	2
GII.13[P21]	1	0	0	0	1
GII.14[P7]	0	0	0	1	1
GIX.1[P15]	1	0	0	1	2
GII.17[P17]	13	0	0	5	18
GII.17[P31]	2	0	0	0	2
GII untypeable	14	0	0	0	14
GI.1[P1]	1	0	0	0	1
GI.2[P2]	10	0	0	1	11
GI.3[P13]	4	0	1	1	6
GI.5[P12]	2	0	0	0	2
GI.6[P11]	5	0	0	1	6
Multiple	6^a^	1	5	2	14
Unknown	63	1	0	8	72
Total	430	13	6	107	556

GII.2[P16] outbreaks predominated across all childcare centers and school settings (Appendix Table). GII.3[P12] outbreaks occurred in childcare centers (19 [6.8%] of 280), primary schools (3 [1.9%] of 155), and middle schools (1 [1.6%] of 61). GII.17[P17] outbreaks occurred in universities (2 [8.3%] of 24), middle schools (4 [6.6%] of 61), primary schools (7 [4.5%] of 155), and childcare centers (2 [0.7%] of 280). GI.2[P2] outbreaks were most commonly detected in middle schools (5 [8.2%] of /61) (χ^2^ 12.907; p = 0.002) (Appendix Table). Of the 14 outbreaks with samples containing multiple genotypes, 4 occurred in universities, 6 in middle schools, 1 in a primary school, and 3 in childcare centers (Appendix Table).

## Discussion

CaliciNet China was launched in October 2016 for the surveillance of norovirus outbreaks in China. By using more sensitive inclusion criteria for reporting acute gastroenteritis outbreaks than PHEESS, CaliciNet China captured more norovirus outbreaks, especially in the catchment area where the network laboratories are located. In addition, the participating laboratories implemented standardized genotyping protocols, which enabled comparison across jurisdictions of norovirus sequences, including emerging strains. The sequence data are accessible only within CaliciNet China.

As found in studies in other countries in the Northern Hemisphere, we found the number of norovirus outbreaks was highest during October–March (*17*,*18*). Person-to-person transmission was the dominant route (95.1%), even higher than has been reported in other countries (*11*,*19*). Almost all (93.7%) outbreaks in our study occurred in childcare centers and schools (primary schools, middle schools, and universities). These outbreak settings have likewise been identified in other Asia countries, such as Japan and South Korea (*18*,*20*), as the most common settings for norovirus outbreaks. In contrast, in the United States and Europe, healthcare facilities (primarily nursing homes and hospitals) are the most commonly reported setting for norovirus outbreaks (*11*,*21*,*22*). The high proportion of norovirus outbreaks in childcare centers and schools seems unique to China and might be associated with the high population density in these settings (*23*) and the enhanced monitoring and reporting of any outbreaks in schools in China. In 2006, the national government started to require school officials to check and screen children attending kindergarten, primary school, and middle schools each morning for fever, vomiting, or diarrhea (*24*). This program has helped facilitate the detection and reporting of infectious diseases, including norovirus, at kindergartens and schools. However, we received few norovirus outbreak reports from other settings involving more adults and elderly persons (such as companies, restaurants, and nursing homes). This difference in the collection of norovirus outbreaks between school and other settings may cause potential bias of the strain patterns of norovirus.

Previous data from CaliciNet and NoroNet have suggested a correlation of certain genotypes with different transmission modes and outbreak settings. Specifically, GII.4 viruses have been associated with person-to-person transmission, whereas non-GII.4 viruses, such as GI.3, GI.6, GI.7, GII.3, GII.6, and GII.12, are more often associated with foodborne transmission (*25*). Other studies reported that GI genotypes more frequently caused waterborne transmission than GII genotypes (*26*). In our study, all outbreaks caused by GII.4 Sydney[P31] were transmitted from person to person; however, GII.2[P16] viruses predominated among both person-to-person and foodborne outbreaks. A recent study also reported that GII.2 outbreaks were transmitted from person to person (77.8%) and food (17.5%) (*27*). The outbreaks caused by multiple genotypes were more often associated with waterborne transmission than foodborne or person-to-person transmission.

The dominant genotype in our study, GII.2[P16], was mainly associated with outbreaks in childcare centers and schools, consistent with a study in Japan, which found GII.2 was the most prevalent genotype in childcare facilities and schools for multiple years (*28*). In other parts of the world, GII.4 viruses have been reported as the dominant genotype among adults and elderly persons, especially in outbreaks in long-term care facilities (*29*,*30*). Our study had only 11 GII.4 outbreaks, and they mainly occurred in childcare centers and primary schools. GII.3[P12] mainly occurred among children in this study, similar to other studies where GII.3 was one of the most common genotypes associated with sporadic norovirus infection, particularly among children (*29*,*31*–*34*). We also found that GII.17 outbreaks tended to infect older children and adults rather than younger children. Previous reports indicated that the median age of persons infected by GII.17 (49, range 9–75 years) was significantly higher than that of GII.4 cases (1, range 1–8 years) (*35*).

During winter 2014–15, GII.17 was reported as the predominant norovirus strain in China, accounting for 67.2% of outbreaks, whereas during the following winter (2015–16), the proportion of GII.17 outbreaks decreased to 25.0% (*36*). We found that GII.2[P16] viruses caused an increase in the number of norovirus outbreaks during winter 2016–17. The first GII.2[P16]–positive sample was detected in August 2016 in Guangdong Province (*36*,*37*), and the number subsequently increased, with GII.2[P16] accounting for 70%–100% of norovirus outbreaks during winter 2016–17 in different provinces (*38*–*40*). Previous studies suggested that reemerging GII.2[P16] virus most likely evolved from strains emerging during 2012–2013 (*41*). Although the antigenicity and histo-blood group antigen binding profile of the early 2016–2017 and pre-2016 GII.2 noroviruses were similar, 1 GII.2[P16] strain with single Val256Ile mutation and the conventionally orientated Asp382 in VP1 showed an expanded histo-blood group antigen binding spectrum in China (*41*). Unlike previously emergent GII.4 viruses, GII.2[P16] did not become a globally predominant genotype; however, this emerging virus was also detected in other countries, such as Germany, Italy, Japan, France, and the United States (*42*–*44*).

Since 2002, GII.4 has been the predominant norovirus genotype in outbreaks in many countries, and every 2–4 years, new GII.4 variants have emerged, displacing previous variants, several of which have been associated with increased norovirus outbreaks worldwide (*8*). Previous studies indicated that global epidemics, including in China, were caused by GII.4 Den Haag (2006b) variant in 2006–2007, New Orleans variant in 2009–2010, and GII.4 Sydney variant in 2012–2013. Although GII.4 Sydney has predominated globally, non-GII.4 strains (GII.17[P17], and GII.2[P16]) have caused 2 recent norovirus epidemics in China (*36*,*45*). These observations highlight the need for enhanced global surveillance for potential epidemics of emerging norovirus genotypes, which may have different regional impacts.

CaliciNet China uses database scripts provided by CaliciNet USA (*16*) and updated detection and dual genotyping protocols (*14*). The dual-typing method is helpful in clarifying the molecular epidemiology of noroviruses, including the identification of newly emerging recombinant viruses. For example, GII.4 Sydney[P31] predominated since it was first recognized in 2012 and subsequently became one of the most successful genotypes causing epidemics globally (*8*). In November 2015, this virus was gradually, although not completely, replaced by a new recombinant GII.4 virus, GII.P16-GII.4 Sydney (*7*), and has since caused ≈50% of all norovirus outbreaks in the United States (https://www.cdc.gov/norovirus/reporting/calicinet/data.html).

Our study has several limitations. First, participation in the network was voluntary, so the distribution of the network does not represent the entire country. Currently, network laboratories are located primarily in eastern and southern China, which generally have better infrastructure. Second, the epidemiologic information for each outbreak collected by CaliciNet China is still limited; however, China CDC is making efforts to include more epidemiologic staff in the network and collect more complete and accurate epidemiologic data. Third, the timeliness of the data reporting in CaliciNet needs to be improved. For a variety of reasons, not all network laboratories were able to submit data to China CDC on a monthly basis. In the future, submission of epidemiologic and laboratory data through a Web-based information system will enable uploading of data in near real time.

CaliciNet China’s use of the same protocols as other norovirus surveillance networks, such as CaliciNet USA, enables comparison of data internationally and potentially provides an early warning when new strains emerge that have the potential to cause global epidemics. In addition, monitoring changes in the distribution of genotypes can help inform the development and assessment of norovirus vaccines, several of which are in clinical trials (*46*,*47*).

In conclusion, we collected information about 556 norovirus outbreaks using standardized epidemiologic definitions and laboratory testing procedures in 6 provinces in China. Person-to-person was the predominant transmission route, and childcare centers and schools were the most common settings for reported norovirus outbreaks. The large number of outbreaks during winter 2016–17 was attributable at least in part to the emergence of the new recombinant genotype GII.2[P16]. CaliciNet China provides essential information about the evolving strain distribution and epidemiologic characteristics of norovirus outbreaks, which can contribute to the development of effective vaccines.

Appendix Genotype setting distribution of norovirus outbreaks reported to CaliciNet China, October 2016–September 2018.
