# Supplementary material for: Norovirus Outbreak Surveillance, China, 2016–2018
Source: Emerg Infect Dis. 2020 Mar;26(3):437–45. doi: 10.3201/eid2603.191183 (PMC7045832; doi:10.3201/eid2603.191183)
Supplement: Appendix — Genotype setting distribution of norovirus outbreaks reported to CaliciNet China, October 2016–September 2018. [file 19-1183-Techapp-s1.pdf]

Article DOI: https://doi.org/10.3201/eid2603.191183

Norovirus Outbreak Surveillance, China, 2016–2018

Appendix

Appendix Table. Genotype setting distribution of norovirus outbreaks reported to CaliciNet China, October 2016–September 2018

	No. (%) outbreaks											
Genotype	Total	Childcare center	Primary school	Middle school	University	Party	Hospital	Company	Hotel	Restaurant	Other*	Unknown
GII.1[P33]	1 (0.2)	0	1 (<0.1)	0	0	0	0	0	0	0	0	0
GII.2[P2]	2 (0.4)	1 (<0.1)	0	1 (1.6)	0	0	0	0	0	0	0	0
GII.2[P16]	349 (62.8)	184 (65.7)	103 (66.4)	35 (57.4)	13 (54.2)	1 (100)	1 (33.3)	1 (11.1)	1 (50.0)	1 (25.0)	8 (50.0)	1 (100)
GII.3[P12]	25 (4.5)	19 (6.8)	3 (1.9)	1 (1.6)	0	0	0	0	0	1 (25.0)	1 (6.3)	0
GII.4 Sydney[P31]	11 (2.0)	4 (1.4)	4 (2.6)	1 (1.6)	1 (4.2)	0	0	1 (11.1)	0	0	0	0
GII.6[P7]	16 (2.8)	8 (2.8)	6 (3.8)	0	0	0	0	2 (22.2)	0	0	0	0
GII.8[P8]	2 (0.4)	1 (<0.1)	1 (<0.1)	0	0	0	0	0	0	0	0	0
GII.13[P21]	1 (0.2)	1 (<0.1)	0	0	0	0	0	0	0	0	0	0
GII.14[P7]	1 (0.2)	0	1 (<0.1)	0	0	0	0	0	0	0	0	0
GIX.1[P15]	2 (0.4)	1 (<0.1)	1 (<0.1)	0	0	0	0	0	0	0	0	0
GII.17[P17]	18 (3.2)	2 (<0.1)	7 (4.5)	4 (6.6)	2 (8.3)	0	0	3 (33.3)	0	0	0	0
GII.17[P31]	2 (0.4)	1 (<0.1)	0	0	0	0	0	0	1 (50.0)	0	0	0
GI.1[P1]	1 (0.2)	0	1 (<0.1)	0	0	0	0	0	0	0	0	0
GI.2[P2]	11 (2.0)	1 (<0.1)	3 (1.9)	5 (8.2)	1 (4.2)	0	0	0	0	0	1 (6.3)	0
GI.3[P13]	6 (1.1)	0	3 (1.9)	1 (1.6)	1 (4.2)	0	0	0	0	0	1 (6.3)	0
GI.5[P12]	2 (0.4)	2 (<0.1)	0	0	0	0	0	0	0	0	0	0
GI.6[P11]	6 (1.1)	2 (<0.1)	1 (<0.1)	1 (1.6)	1 (4.2)	0	0	0	0	0	1 (6.3)	0

	No. (%) outbreaks											
Genotype	Total	Childcare center	Primary school	Middle school	University	Party	Hospital	Company	Hotel	Restaurant	Other*	Unknown
Multiple	14 (2.5)	3 (0.1)	1 (<0.1)	6 (9.8)	4 (16.7)	0	0	0	0	0	0	0
GII untypeable	14 (2.5)	9 (3.2)	3 (1.8)	1 (1.6)	0	0	0	0	0	0	1 (6.3)	0
Not determined†	72 (12.9)	41(14.6)	16 (10.3)	5 (8.2)	1 (4.2)	0	2 (66.7)	2 (22.2)	0	2 (50.0)	3 (18.7)	0
Total	556	280	155	61	24	1	3	9	2	4	16	1

*Fifteen outbreaks occurred in schools that combine primary school and middle school, and 1 outbreak occurred in a school that combines a childcare center, a primary school, and a middle school.

†The genotyping reverse transcription PCR for these outbreaks was not conducted by network laboratories.

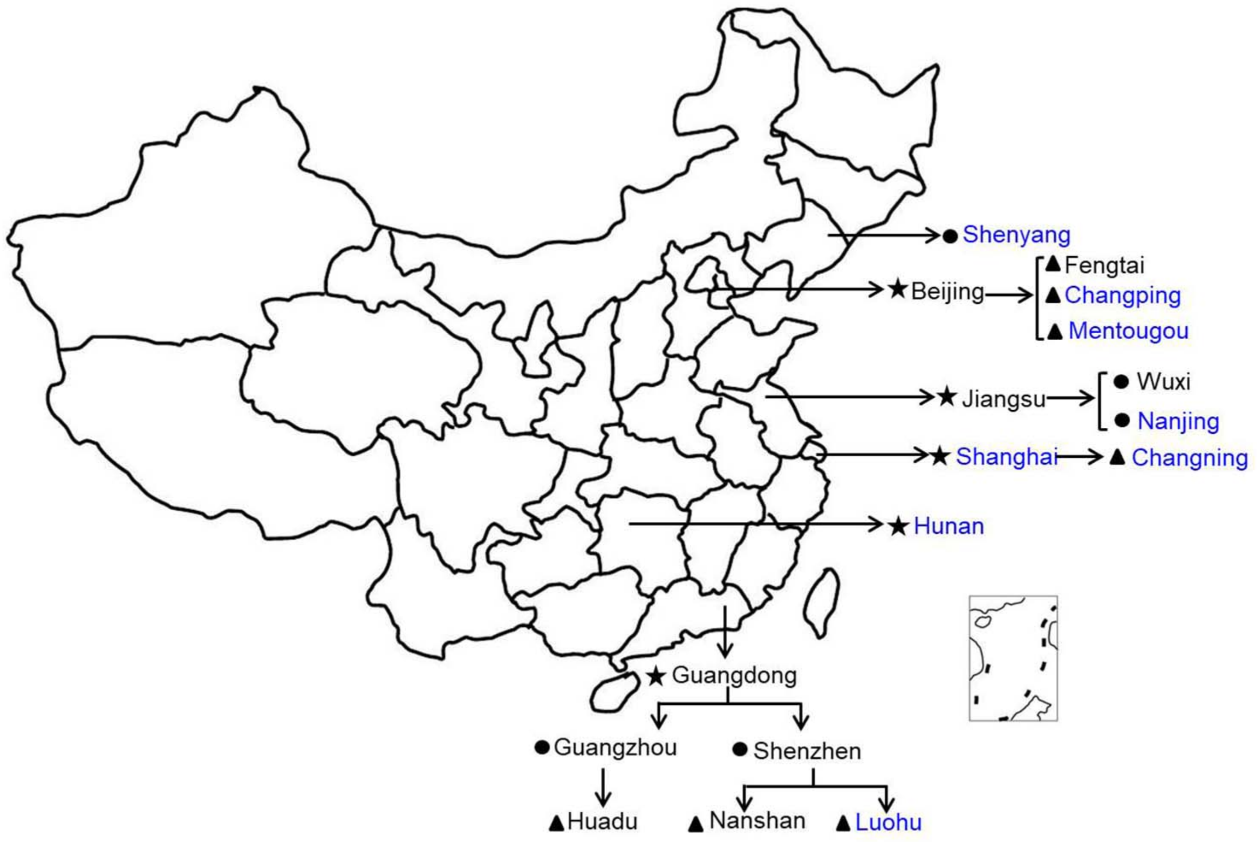

Figure 1. Geographic location of participating local Centers for Disease Control and Prevention in CaliciNet China, October 2016-September 2018. Star indicates provincial/municipality laboratories; circle, city laboratories; triangle, district/county laboratories. Laboratories that participated in CaliciNet China: black, April 2016; blue, April 2017.
